# Difference in CXCR4 expression between sporadic and VHL-related hemangioblastoma

**DOI:** 10.1007/s10689-016-9879-3

**Published:** 2016-02-26

**Authors:** Roeliene C. Kruizinga, Denise M. S. van Marion, Wilfred F. A. den Dunnen, Jan C. de Groot, Eelco W. Hoving, Sjoukje F. Oosting, Hetty Timmer-Bosscha, Rosalie P. H. Derks, Chantal Cornelissen, Rob B. van der Luijt, Thera P. Links, Elisabeth G. E. de Vries, Annemiek M. E. Walenkamp

**Affiliations:** 1 Department of Medical Oncology, University Medical Center Groningen, University of Groningen, P.O. Box 30.001, 9700 RB Groningen, The Netherlands; 2 Department of Endocrinology, University Medical Center Groningen, University of Groningen, P.O. Box 30.001, 9700 RB Groningen, The Netherlands; 3 Department of Pathology, University Medical Center Groningen, University of Groningen, P.O. Box 30.001, 9700 RB Groningen, The Netherlands; 4 Department of Radiology, University Medical Center Groningen, University of Groningen, P.O. Box 30.001, 9700 RB Groningen, The Netherlands; 5 Department of Neurosurgery, University Medical Center Groningen, University of Groningen, P.O. Box 30.001, 9700 RB Groningen, The Netherlands; 6 Department of Medical Genetics, University Medical Center Utrecht, P.O. Box 85.500, 3508 GA Utrecht, The Netherlands

**Keywords:** CXCR4, CXCL12, Hemangioblastomas, VEGF, VHL

## Abstract

Central nervous system hemangioblastomas occur sporadically and in patients with von Hippel–Lindau (VHL) disease due to a *VHL* germline mutation. This mutation leads to enhanced transcription of chemokine receptor 4 (CXCR4), its ligand (CXCL12) and vascular endothelial growth factor A (VEGFA). We aimed to determine in VHL-related and sporadic hemangioblastomas CXCR4, CXCL12, and VEGFA protein expression and to correlate this to hemangioblastoma size and expression in normal surrounding tissue. 27 patients with a hemangioblastoma were included for analysis of immunohistochemistry of tissue, MRI and DNA. Hemangioblastomas overexpress CXCR4, CXCL12, and VEGFA compared to normal surrounding tissue. In sporadic hemangioblastomas the mean percentage of CXCR4 positive hemangioblastoma cells was 16 %, SD 8.4, in VHL-related hemangioblastomas 8 %, SD 4.4 (*P* = 0.002). There was no relation between preoperative tumor size and CXCR4 or CXCL12 expression. Compared to normal surrounding tissue CXCR4, CXCL12, and VEGFA were overexpressed in hemangioblastomas. Most interestingly, sporadic hemangioblastomas overexpress CXCR4 compared to VHL-related hemangioblastoma.

**Electronic supplementary material:**

The online version of this article (doi:10.1007/s10689-016-9879-3) contains supplementary material, which is available to authorized users.

## Introduction

von Hippel–Lindau (VHL) disease is an autosomal dominant syndrome leading to the onset of multi-organ benign and malignant neoplasms including hemangioblastomas. Hemangioblastomas are highly vascularized lesions comprising 1.1–2.4 % of central nervous system (CNS) neoplasms. Their principal components are hemangioblastoma stromal cells with a low mitotic index and capillaries formed by normal endothelial cells with activated pericyte coverage. Approximately a third of hemangioblastomas is related to VHL-disease [1]. However, this might be an underestimation as not all cases are screened for germ-line VHL-mutations.

The characteristic vascularized appearance of hemangioblastomas in VHL-disease patients is thought to be caused by the VHL-mutation leading to defective or absent VHL protein (pVHL). Improper functioning pVHL fails to degrade hypoxia inducible factor 1A (HIF1A) thereby resulting in increased transcription of proteins such as platelet-derived growth factor, transforming growth factor A and vascular endothelial growth factor (VEGFA) [2–4]. Upon expression VEGFA is released into the environment by the tumor cells and binds to its corresponding receptors mainly on endothelial cells. In this way VEGFA primarily induces endothelial cell proliferation and migration, promoting the aberrant angiogenesis that supports tumor growth and tumor metastasis [5].

Several in vitro and in vivo models have revealed a key role of chemokine receptor 4 (CXCR4) and its ligand CXCL12 in the crosstalking between cancer cells and their microenvironment [6, 7]. CXCR4 is downregulated by the VHL protein and upregulated by HIF1A. CXCR4 and CXCL12 are co-expressed in CNS hemangioblastoma and in renal clear cell carcinoma [8]. Large tumors have more risk on hypoxic conditions. Increased hypoxia leads to higher CXCR4 expression via HIF1α. Loss-of-function of the *VHL* gene is responsible for the upregulation of the expression of CXCR4, its ligand CXLC12, and VEGFA. This is particularly interesting as these micro-environmental factors are increasingly recognized and established as novel drug targets [9].

Therefore, the aim of this study was to investigate CXCR4, CXCL12, and VEGFA protein expression in VHL-related and sporadic hemangioblastomas and to correlate this to size as measured by MRI before surgery to investigate possible differences between VHL-related and sporadic hemangioblastoma. In order to verify the contribution of somatic *VHL* mutations and hypermethylation of hemangioblastomas we analyzed *VHL* mutations and promoter methylation in the tumor tissue.

## Materials and methods

### Patients

All patients that were operated between 1995 and 2010 in the University Medical Center Groningen from who frozen hemangioblastoma tissue was available in the tissue bank of the Department of Pathology were eligible. Patients without a known germline mutation were classified as having sporadic hemangioblastoma and patient with a known germline mutation were as having VHL-related hemangioblastoma. All patients apart from one, who refused screening for germline *VHL*-mutation, were routinely tested for mutations.

Hemangioblastoma tissues were numerically tagged according to a national coding system. Clinical data obtained from VHL surveillance were used to retrieve data on gender and age. Lesion size of both solid nodule and associated cyst size were based on pre-operative MRI scan and on written surgery reports. According to the VHL guidelines, MRI scans from the cerebellum and myelum were performed biennially [10–13], annually when a lesion was present, and every 6 months when a lesion was found to have increased in size until stable disease was ascertained (www.stoet.nl, VHL working group). The largest diameter in mm was used and surface area in mm^2^ was then calculated. Solid tumor size was defined by the diameter of only the solid nodule and cyst size by only the cystic part. Total size was defined by largest diameter of both solid and cystic part together. All data was stored in a computerized anonymous database. Patient identity was protected by unique codes. The Medical Ethical Review Board declared that based on Dutch law no further approval for use of residual tissue for analysis, including DNA analysis, was needed.

### Immunohistochemistry

Cryosections (4 μm) were morphologically defined as hemangioblastoma by hematoxylin and eosin (H&E) staining. For CXCR4, CXCL12, and VEGFA staining, the fixation procedure was followed by endogenous peroxidase blockage by 1 % (w/v) hydrogen peroxidase. CXCR4 was stained in dilution 1:500 (polyclonal rabbit #ab2074, Abcam, Cambridge, UK) and CXCL12 in dilution 1:60 (monoclonal mouse #MAB350, R&D Systems, Abingdon, UK). VEGFA staining was performed using the avidin–biotin complex method (avidin–biotin blocking kit, Vector Laboratories, Peterborough, UK) with anti-VEGFA as primary antibody in dilution 1:50 (polyclonal rabbit #sc-152, Santa Cruz, Heidelberg, Germany). All secondary and tertiary goat anti rabbit horse radish peroxidase (HRP), rabbit anti goat HRP and rabbit anti mouse HRP, secondary swine anti rabbit biotinylated antibodies and streptavidin-HRP were obtained from Dako (Glostrup, Denmark). Frozen sections of glioblastoma (WHO grade 4) served as positive controls for CXCR4 and VEGFA. Cytospins of renal cell carcinoma cells (RCC786, authenticated by STR analysis) served as the positive controls for CXCL12. For negative controls, the primary antibodies were omitted.

Normal surrounding brain tissue, small fragments of preexistent normal brain parenchyma excised during debulking surgery, was verified by morphology on H&E staining. CXCR4 quantitative evaluation was performed by counting the percentage of positive cells (of total cells) in 5 random, but qualitatively good, high power fields (400× magnification) per hemangioblastoma slide. CXCL12 semi quantitative evaluation was performed in 5 high power fields (400× magnification) per slide and defined as negative, positive (>1 % positive cells) or strongly positive (>50 % positive cells or high intensity). VEGFA was evaluated by describing morphology of tissue (normal brain tissue, vascular endothelium and stromal hemangioblastoma cells) in relation to positive cells. Evaluation and quantification was performed by two independent observers (RCK and NK) blinded to the diagnosis, using NDP software (Hamamatsu, Almere, the Netherlands) after scanning slides with a Hamamatsu scanner (Hamamatsu).

### DNA isolation

For DNA isolation frozen hemangioblastoma scrapings (10 times 10 μm) were collected from all 33 hemangioblastomas. Also 2 male and 2 female samples of normal tissue were collected. DNA was extracted using dissolving buffer [4 mol/l guanidine thiocyanate-buffer, DNase free water, 96 % ethanol and further digested in Tris EDTA (TE)-buffer (10 mmol/l Tris/HCl, pH = 7.5, 1 mmol/l EDTA with proteinase K and sodium dodecyl sulfate 10 % (w/v))]. Standard salt extraction and isopropanol precipitation was used for high molecular DNA and dissolved in 150 µl TE-buffer. For quality control, genomic DNA was amplified in a multiplex PCR according to the BIOMED-2 protocol [14].

### Sequencing and multiplex ligation-dependent probe amplification (MLPA)

Germline mutations were defined by inherited mutations in *VHL* or inherited loss of the whole gene as found in loss of heterozygosity (LOH) analysis. Exons 1, 2 and 3 of *VHL* and their flanking sequences were amplified by PCR. PCR products were purified and subjected to sequence analysis using an ABI 3730 automated DNA sequencer (Applied Biosystems, Life Technologies Corporation, CA, USA). To detect genomic deletions involving single or multiple exons of *VHL*, an MLPA-assay (MRC-Holland SALSA MLPA P016 VHL probe mix, Amsterdam, the Netherlands) was performed according to the manufacturer’s instructions. For the interpretation of sequence alterations, the Alamut (Interactive Biosoftware, Rouen, France) decision-support software package was used.

### Methylation specific PCR (MSP)

MSP was performed after bisulphite treatment on denatured genomic DNA. Bisulphite treatment was performed with the EZ DNA methylation kit according the manufacturer’s protocol (Zymogen, BaseClear, Leiden, the Netherlands). For PCR 15 ng of bisulphite treated DNA was used. The *β*-*actin* gene served as an internal reference. Primer pair sequences are listed in Supplementary Table 2 (designed with Methyl Primer Express v1.0, Invitrogen). PCR products were visualized on a 2.5 % (w/v) agarose gel. A sample was considered positive (methylated or hypermethylated) when a PCR product of the right size was visible after 40 cycles of PCR. Leukocyte DNA collected from anonymous healthy volunteers and in vitro CpG methylated DNA with SssI (CpG) methyltransferase (New England Biolabs Inc., Beverly, MA, USA) were used as negative and positive control, respectively.

### Statistical analysis

Statistical analysis was done using Kruskal–Wallis test, linear regression and Spearman rank correlation. *P* values of <0.05 were considered significant.

## Results

Thirty-three specimens of 27 patients operated between 1995 and 2010 for central nervous system hemangioblastoma were analyzed. Sixteen specimens were from 11 VHL-disease patients (seven with 1, three with 2 and one patient with 3 specimens) and 17 specimens from 16 sporadic cases (15 with 1 and one with 2 specimens) (Table 1).

**Table 1:** Characteristics of hemangioblastoma patients and their tissues

Characteristic	No of tissues (%)	No of patients (%)	Age at time of surgery in years (range)
Sex			
Male	21 (64 %)	17	47 (13–62)
Female	12 (36 %)	10	41 (14–65)
*VHL*-mutation carriers	16 (48 %)	11 (41 %)	46 (26–65)
c.259_260-insA p.Val87Aspfs*45	1 (6 %)		
c.-89-?_c297+?del. p(?)	7 (44 %)		
c.341-59_341-14del p.?	1 (6 %)		
c.462A>C p.Pro154Pro	1 (6 %)		
c.500G>A p.Arg167Gln	3 (19 %)		
c.463+2T>C p.(?)	1 (6 %)		
c.490C>T p.Gln164*	2 (13 %)		
Sporadic	17 (52 %)	16 (59 %)	44 (13–62)

**Fig. 1:**
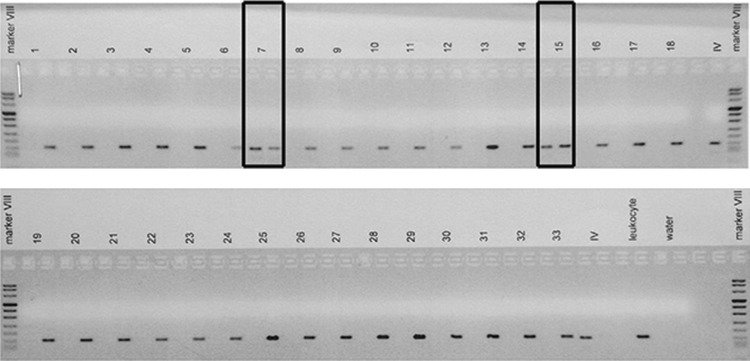
All *VH*L methylation specific PCR (MSP) products, 33 hemangioblastoma tissues (numbered 1–17 sporadic cases, 18–33 VHL related cases) and controls (in vitro CpG methylated DNA with Sssl CpG methyltransferase (IV), leukocyte and water), on agarose gel. From each sample the first lane is the methylated DNA and the second lane the unmethylated DNA. Hemangioblastoma derived DNA sample 7 and 15 (*boxes*) show methylation of the *VHL*-promoter, both these samples were derived from sporadic hemangioblastoma patients. Experiments were performed in triplicate

In 27 specimens sufficient DNA was available to perform mutation analysis (Table 2, Supplementary Table 1). In 76.9 % of cases (10 out of 13) of VHL-related hemangioblastomas only the germline mutation was found, in 15.4 % (2 out of 13) two mutations were present (second hit) and in one case 3 mutations were found. In the sporadic hemangioblastomas, 57 % (8 out of 14) were mutated. In two out of 27 hemangioblastomas specimens the VHL promoter was hypermethylated, both were sporadic hemangioblastoma cases ( Fig. 1).

**Table 2:** Occurrence of *VHL*-mutations and CXCR4 expression in the hemangioblastoma (HB) specimens

	VHL-related HB		Sporadic HB	
	Percentage (number)	CXCR4 expression (% post. cells)	Percentage (number)	CXCR4 expression (% post. cells)
Two normal alleles	–	–	43 (6/14)	17.0
One mutation	76.9 (10/13)	8.2	14.3 (2/14)	16.5
Two mutations	15.4 (2/13)	10.2	7.1 (1/14)	16.1
LOH	0	–	21.4 (3/14)	24.5
One mutation and LOH	0	–	14.3 (2/14)	12.1
Three mutations	7.7 (1/13)	7.3	0	–

### CXCR4, CXCL12 and VEGFA expression

**Fig. 2:**
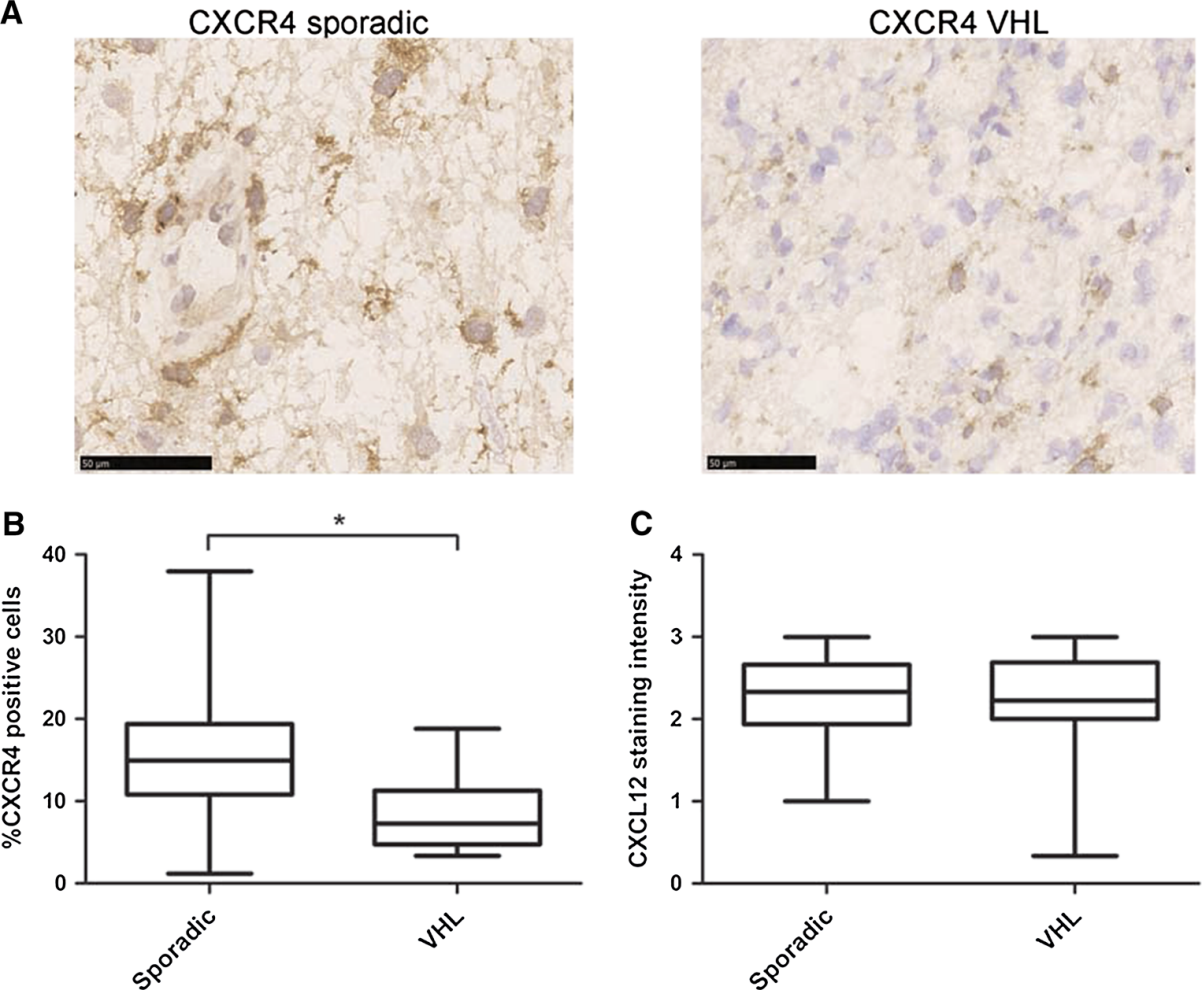
**a** Representative pictures CXCR4 immunohistochemistry (×40 magnification) on sporadic (*left*) and VHL-disease related (*right*) hemangioblastoma specimens. CXCR4 expression is present in all hemangioblastoma cells, excluding endothelial cells [vessels are depicted by an *asterisk* (*)]. **b**, **c** *Box plots* of percentage of CXCR4 positive cells (**b**) and CXCL12 staining intensity (**c**) per field of view in sporadic and VHL-disease related hemangioblastoma specimens showing a higher mean percentage of CXCR4 positive cells but similar CXCL12 expression in sporadic hemangioblastoma compared to VHL-related hemangioblastoma. Linear regression, *P < 0.05. The *bars* represent the range

All hemangioblastoma cells, excluding endothelial cells, showed CXCR4 expression (n = 29, as 4 slides were not evaluable; Fig. 2A). In the 16 sporadic hemangioblastomas the mean percentage of CXCR4 positive cells per specimen (16 %, range 1–38 %) was higher as compared to the 13 VHL-related hemangioblastomas (8 %, range 3–19 %, *P* = 0.002; Fig. 2b; Table 2). In the 15 available normal surrounding brain tissue samples no CXCR4 expression was observed.

**Fig. 3:**
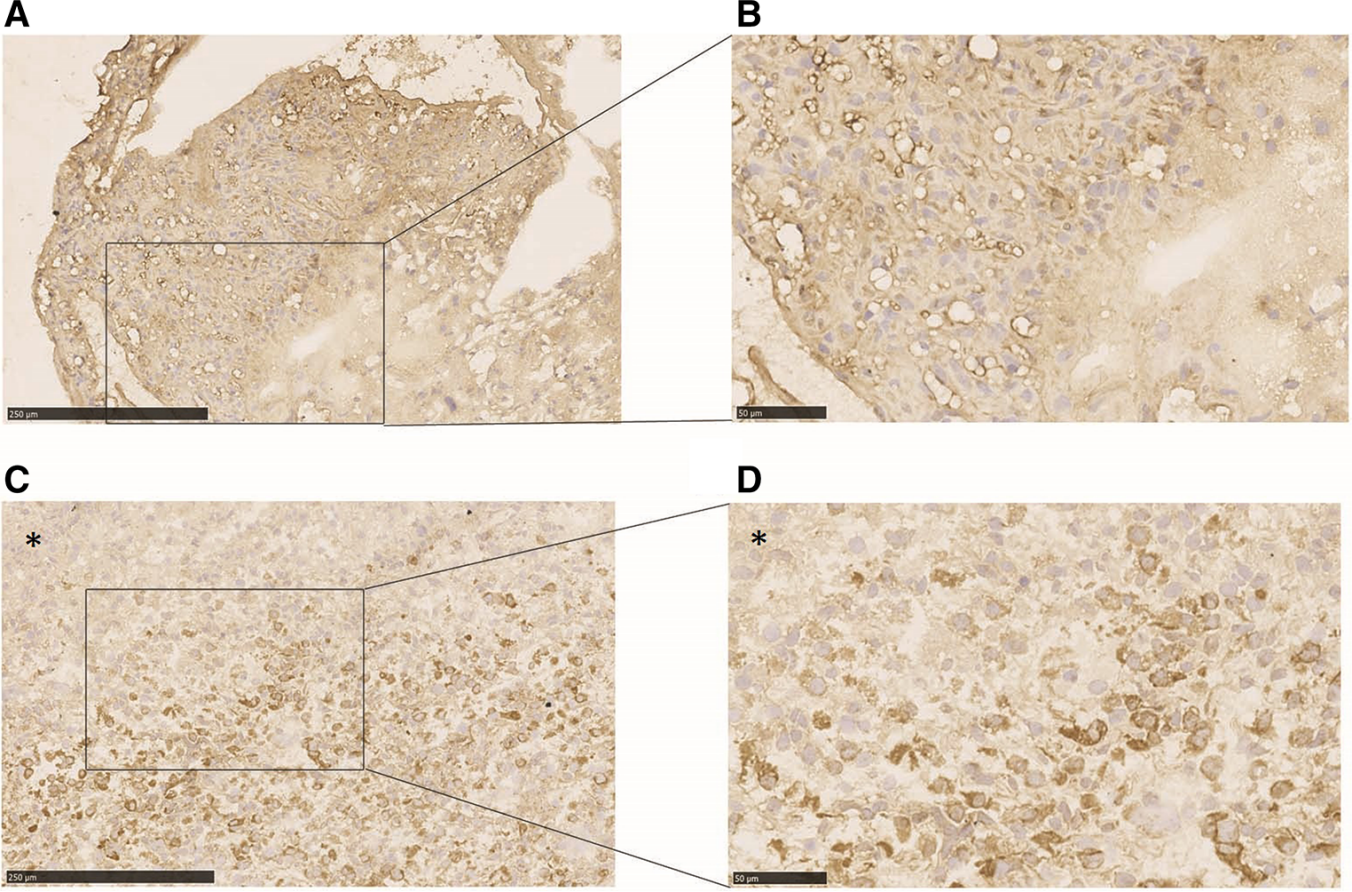
Hemangioblastoma tissue overexpresses CXCL12 compared to normal tissue as depicted in the representative pictures of CXCL12 immunohistochemistry on VHL-disease (**a** ×40 magnification and **b** computer magnification) and hemangioblastoma (**c** ×40 magnification and **d** computer magnification) specimens [normal tissue within hemangioblastoma specimen depicted by an *asterisk* (*)]

**Fig. 4:**
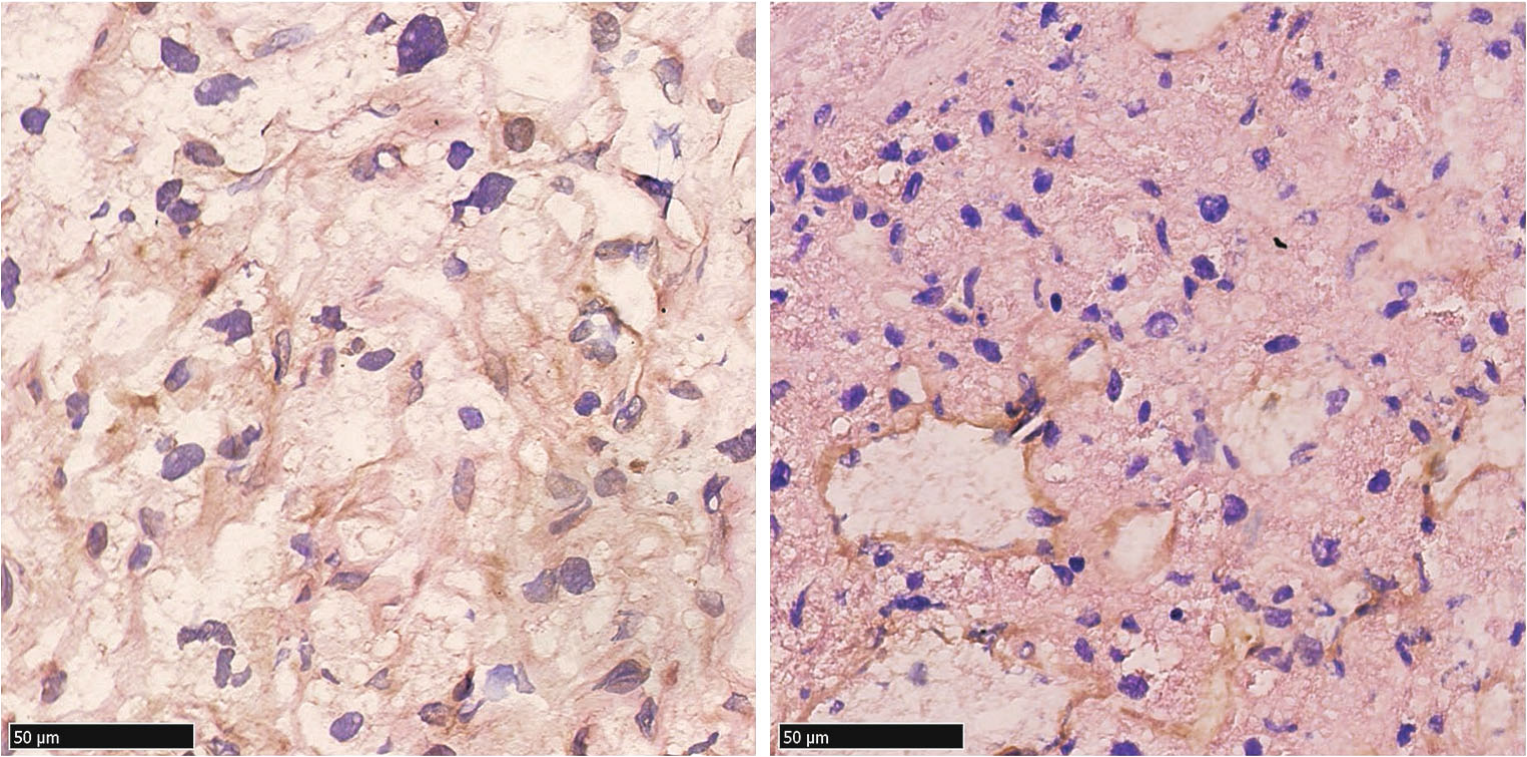
Stromal hemangioblastoma cells and vascular endothelial cells show higher immunohistochemical (×40 magnification) VEGFA expression in sporadic (*left*) and VHL-related (*right*) than normal tissue in hemangioblastoma specimens

Thirty two samples were evaluable for CXCL12 expression. Sporadic and VHL-related hemangioblastomas had the same level of CXCL12 expression (Fig. 2c), with strong expression in 75 % (12 out of 16) of sporadic hemangioblastomas and in 81 % (13 out of 16) of VHL-related hemangioblastoma cells. The normal tissue showed no (n = 12) or in a few cases (n = 3) some expression of CXCL12 (Fig. 3). VEGFA was expressed higher than normal in sporadic and VHL-related hemangioblastomas, and present in stromal hemangioblastoma cells and vascular endothelial cells (Fig. 4).

### Correlation between CXCR4 expression and clinical variables

**Fig. 5:**
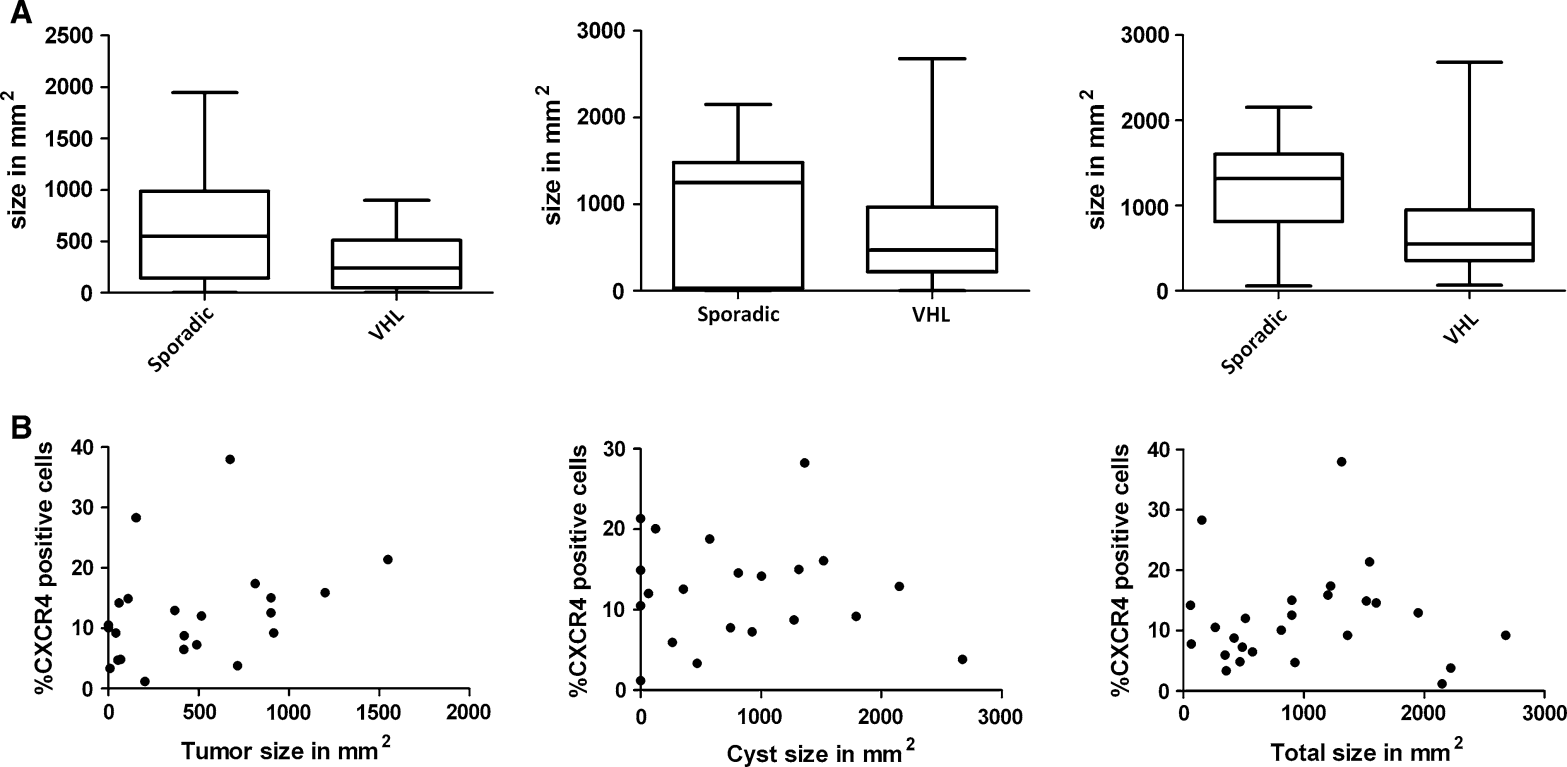
**a** Boxplots of size in mm^2^ of solid tumor (*left*), cyst (*middle*) and largest total diameter (*right*) of the sporadic and VHL-disease related hemangioblastoma specimens, measured on pre-operative MRI images (*bars* represent the range). Hemangioblastomas and associated cyst size was similar for sporadic and VHL-related hemangioblastoma specimens. **b** Solid tumor (*left*), cyst (*middle*) and total (*right*) hemangioblastoma size in mm^2^ and the percentage of CXCR4 positive cells per field of view measured by CXCR4 immunohistochemistry were not correlated

Sporadic hemangioblastomas and associated cyst size did not differ from VHL-related hemangioblastomas (sporadic: median solid tumor size 547.5 mm^2^, median total size 1316.3 mm^2^ and VHL-related: median solid tumor size 235.75 mm^2^, and median total size 545.88 mm^2^, *P* = 0.09 for both; Fig. 5).

The tumor and associated cyst size was not correlated to the CXCR4 expression level (95 % confidence interval 298.8; 700.1 mm^2^ rho = 0.39, *P* = 0.069 for tumor nodule and 95 % confidence interval 763.7; 1295.5 mm^2^ rho = 0.095, *P* = 0.644 for total size; Fig. 5).

## Discussion

This study shows that CXCR4, CXCL12, and VEGFA are all overexpressed in stromal hemangioblastoma cells compared to normal surrounding brain tissue. This is the first study in which expression of CXCR4, CXCL12, and VEGFA is compared in both sporadic and VHL associated hemangioblastoma patients. Earlier studies in VHL related hemangioblastoma found upregulated CXCR4, CXCL12, and VEGFA. In contrast, studies in VHL-related renal cell carcinoma and retinal hemangioblastoma high levels of CXCR4 and VEGF, and not CXCL12, were found [15–17]. In the current study CXCL12 and VEGFA expression was similar in sporadic and VHL-related cases.

The finding that CXCR4 expression was higher in sporadic hemangioblastomas is striking as in VHL-disease a germline mutation in *VHL* leads to a defect VHL protein which results in enhanced transcription of CXCR4, its ligand CXCL12 as well as VEGFA [8]. To analyze the possible reason for the observed difference we determined the genetic background of hemangioblastomas of both VHL-related and sporadic cases. The onset of lesion formation in VHL-disease occurs when the inherited germline mutation is accompanied by a second hit, e.g. a mutation in the normal allele. Previous studies reported inactivation of both alleles of *VHL* in 62 of VHL-disease related hemangioblastoma [20]. For VHL-related renal cell carcinoma this percentage is even higher, 86.6 % [21]. In sporadic hemangioblastomas *VHL* is inactivated as well. Others reported that in 20–50 % of cases one allele was inactivated and in 0–13 % both *VHL* alleles [20, 22–27]. Our data show that in all VHL-related and in the majority of sporadic hemangioblastomas a mutation, LOH or hypermethylation of the *VHL* was present. In addition no difference in CXCR4 expression between patients with and without a *VHL*-mutation was found and thus could not explain the observed difference in CXCR4 expression in VHL and sporadic hemangioblastomas.

Next we hypothesized that the size of hemangioblastomas at the time of surgery might explain the observed difference in CXCR4 expression and analyzed MRIs for hemangioblastoma size. Bulky tumors develop more hypoxia then smaller ones. Therefore, bulky tumors, with more hypoxia, are hypothesized to express higher levels of CXCR4, as hypoxia and CXCR4 upregulation are correlated [15]. Hypoxia has been found to be inversely correlated to tumor size in murine models [18, 19]. Although VHL patients are screened by biennial MRI [10–13] we did not find a significant difference in preoperative size of VHL-related and sporadic hemangioblastomas and associated cysts.

We also determined the genetic background of hemangioblastomas of both VHL-related and sporadic cases. The onset of lesion formation in VHL-disease occurs when the inherited germline mutation is accompanied by a second hit, e.g. a mutation in the normal allele. Previous studies reported inactivation of both alleles of *VHL* in 62 of VHL-disease related hemangioblastoma [20]. For VHL-related renal cell carcinoma this percentage is even higher, 86.6 % [21]. In sporadic hemangioblastomas *VHL* is inactivated as well. Others reported that in 20–50 % of cases one allele was inactivated and in 0–13 % both *VHL* alleles [20, 22–27]. Our data show that in all VHL-related and in the majority of sporadic hemangioblastomas a mutation, LOH or hypermethylation of the *VHL* was present. In addition no difference in CXCR4 expression between patients with and without a *VHL*-mutation was found and thus could not explain the observed difference in CXCR4 expression in VHL and sporadic hemangioblastomas.

We and others found CXCR4 overexpressed in hemangioblastoma cells compared to normal surrounding brain tissue [8]. The difference in CXCR4 expression between these two cell types might be caused by their difference in origin, the hemangioblastoma cell is a mesoderm-derived, embryologically arrested hemangioblast [22]. As the CXCR4 expression is limited to hemangioblastoma tissue, this potentially creates opportunities for treatment with drugs specifically targeting CXCR4 expressing cells. Since CXCR4 expression is involved in primary tumor growth [28], tumor invasiveness [29], metastasis [29], angiogenesis [30] and vasculogenesis [31], this subpopulation of CXCR4 overexpressing cells can be targeted by CXCR4 inhibitors. The tumor growth-stimulating role of CXCR4 was confirmed by showing that CXCR4 antagonists inhibit tumor growth in multiple experimental orthotopic, subcutaneous human xenograft, and transgenic mouse models [32]. In a transgenic breast cancer mouse model, treatment with the CXCR4 inhibition CTCE-9908 resulted in a 56 % reduction in primary tumor growth rate compared to controls receiving scrambled protein. Moreover, this coincided with a 42 % reduction in vascular endothelial growth factor (VEGF) protein expression and 30 % reduction in p-AKT/AKT expression [32]. Furthermore, in various cancer models metastasis of cancer cells was revealed to be mediated by CXCR4 activation and directed migration towards CXCL12 expressing organs [33–37]. Experimental metastatic mouse models have provided evidence that targeting CXCR4 impairs cancer cell metastasis [29, 32, 37–42]. The CXCR4 inhibitor AMD3100 is FDA approved for use in patients with non-Hodgkin’s lymphoma and multiple myeloma. It is registered to be used as stem cell mobilizer [43] and is tested as investigational agent as anticancer drug for other cancers [44].

For VHL-related hemangioblastoma, targeted therapy against CXCR4 and VEGFA could potentially be combined with conventional therapy as VHL-related disease is multiple and lesions often reoccur after surgery [45–47]. Besides, as VHL disease is an autosomal dominant multisystem neoplastic syndrome [48, 49] targeted therapy would be a good option to treat VHL disease at other sites, for example (metastasizing) renal cell carcinoma. We also found VEGFA overexpressed in stromal hemangioblastoma cells as compared to normal surrounding brain tissue. Bevacizumab, an anti-VEGFA monoclonal antibody, has been extensively investigated in several settings, ranging from single agent treatment to combined modality approaches in both recurrent and newly diagnosed malignancies [50]. Preclinical experiments show that the combination of AMD3100 and bevacizumab may help prevent the growth of certain tumors like gliomas [51, 52]. In the presence of HIF1α bone marrow-derived CD45 + myeloid cells containing Tie2+, VEGFR1+, CD11b+, and F4/80+ subpopulations, as well as endothelial and pericyte progenitor cells promote neovascularization, party trough increase in CXCL12, the ligand of CXCR4. MMP-9 activity of bone marrow-derived CD45+ cells is essential to initiate angiogenesis by increasing VEGF bioavailability [51]. Impaired pVHL fails to degrade HIF1α resulting in increased VEGFA levels and therefore combined CXCR4 and VEGFA inhibition could be of potential benefit for VHL patients. A phase I study of the CXCR4 inhibitor plerixafor and bevacizumab is ongoing for patients with recurrent high-grade gliomas (NCT01339039). This combination could be potentially of high interest for both VHL related and sporadic patients with hemangioblastoma.

## Conclusions

CXCR4, CXCL12, and VEGFA were all overexpressed in hemangioblastomas as compared to normal surrounding tissue. Sporadic hemangioblastomas express more CXCR4 as compared to VHL-related hemangioblastoma. The difference in CXCR4 expression was not explained by size, or *VHL* mutation and/or hypermethylation. CXCR4 expression in hemangioblastoma tissue potentially creates treatment opportunities with drugs specifically targeting CXCR4 expressing cells.

## Electronic supplementary material

Below is the link to the electronic supplementary material.

Supplementary material 1 (DOC 72 kb)
