# Supplementary material for: Difference in CXCR4 expression between sporadic and VHL-related hemangioblastoma
Source: Fam Cancer. 2016 Feb 26;15(4):607–16. doi: 10.1007/s10689-016-9879-3 (PMC5010837; doi:10.1007/s10689-016-9879-3)
Supplement: Supplementary file 1 — Supplementary material 1 (DOC 72 kb) [file 10689_2016_9879_MOESM1_ESM.doc]

| **Supplementary Table 1.** Results from mutation, LOH and methylation analysis. | | | | | |
| --- | --- | --- | --- | --- | --- |
|  | Patient sample | Mutation | LOH | Hyper-methylation | Overall DNA status |
| **Sporadic** | 1 | c.227_220del  p.Phe76del | Normal | No | Somatic |
|  | 2 | Normal | Normal | No |  |
|  | 3 | Normal | Normal | No |  |
|  | 4 | c.233A>G  p.Asn78Ser | VHL-gene | No | 2 Somatic |
|  | 5 | Normal | VHL-gene | Yes | Somatic |
|  | 6 | Normal | Normal | No |  |
|  | 7 | Normal | Normal | No |  |
|  | 8 | - | - | Yes | Somatic |
|  | 9 | c.208_237dup  p.Glu70_Arg79dup  c.233A>G  p.Asn78Ser | Normal | No | 2 Somatic |
|  | 10 | - | - | No |  |
|  | 11 | Normal | Normal | No |  |
|  | 12 | c.317delG  p.Gly106Alafs*53 | VHL-gene | No | 2 Somatic |
|  | 13 | c.499C>T  p.Arg167Trp | Normal | No | Somatic |
|  | 14 | - | - | No |  |
|  | 15 | Normal | Normal | No |  |
|  | 16.1 | Normal | VHL-gene | No | Somatic |
|  | 16.2 | Normal | VHL-gene | No | Somatic |
| **VHL** | 1 | c.500G>A  p.Arg167Gln | Normal | No | Germline |
|  | 2 | c.341-59_341-14del  p.?  c.408del  p.Phe136Leufs*23 | VHL-gene | No | Germline and 2 somatic |
|  | 3 | c.462A>C  p.Pro154Pro | Normal | No | Germline |
|  | 4 | Normal | VHL-gene  C3ORF10-gene | No | Germline |
|  | 5 | - | - | No |  |
|  | 6 | Normal | VHL-gene  C3ORF10-gene | No | Germline |
|  | 7 | c.259_260insA  p.Val87Aspfs*45 | Normal | No | Germline |
|  | 8.1 | Normal | VHL-gene  C3ORF10-gene | No | Germline |
|  | 8.2 | - | - | No |  |
|  | 9.1 | c.292C>A  p.Tyr98*  c.500G>A  p.Arg167Gln | Normal | No | Germline and somatic |
|  | 9.2 | c.500G>A  p.Arg167Gln | Normal | No | Germline |
|  | 10.1 | c.490C>T  p.Gln164* | Normal | No | Germline |
|  | 10.2 | c.490C>T  p.Gln164* | Normal | No | Germline |
|  | 11.1 | c.463+3A>C  p.? | VHL-gene  C3ORF10-gene | No | Germline and somatic |
|  | 11.2 | - | - | No |  |
|  | 11.3 | Normal | VHL-gene  C3ORF10-gene | No | Germline |
| All hemangioblastoma samples were numbered according their origin; Sporadic or VHL hemangioblastoma, and multiple samples of one patient were assigned by .1, .2, and .3. Mutations in *VHL* are described by the new classification system. The column *Overall* describes per sample the summary of all DNA analyses performed. Samples with not sufficient DNA available for analysis are marked by -. | | | | | |

| **Supplementary Table 2**. Sequence (5’to 3’) primers *VHL*. | | |
| --- | --- | --- |
|  | Forward Primer | Reverse Primer |
| Methylated | GTAGTTTCGTTTCGCGTTC | TTCAAAACCGTACTCTTCGA |
| Unmethylated | GTATGTAGTTTTGTTTTGTGTTT | CTTCAAAACCATACTCTTCAA |
